# Schwannoma with chondroid metaplasia of the external auditory canal – a rare finding in a rare location: a case report

**DOI:** 10.1186/s13256-018-1584-4

**Published:** 2018-03-13

**Authors:** Amal Bennani, Nassira Karich, Imane Kamaoui, Meryem Chraibi, Sanaa Abbaoui

**Affiliations:** 1 Departement of Pathology, Mohamed I University, 30050 Oujda, Morocco; 2 Departement of Radiology, Mohamed I University, 30050 Oujda, Morocco; 3 Assaada, BP 6210, 60020 Oujda, Morocco

**Keywords:** Schwannoma, External auditory canal, Chondroid metaplasia

## Abstract

**Background:**

Schwannomas are uncommon tumors of the external auditory canal. In the English literature, very few cases of schwannomas originating in the external auditory canal were reported and none of them showed chondroid metaplasia. We report the first case of schwannoma with chondroid metaplasia in this location.

**Case presentation:**

In this report, we described a 22-year-old white man who presented with an external auditory slow growing mass. A computed tomography scan of the temporal bone demonstrated a well-circumscribed, soft tissue mass narrowing most of the external auditory canal. A surgical biopsy was performed and the histological examination showed a schwannoma with chondroid metaplasia.

**Conclusion:**

Schwannoma should be considered in the differential diagnosis of benign or malignant tissue masses involving the external ear canal.

## Background

Schwannomas are slow growing benign tumors arising from Schwann cells of peripheral nerve sheaths. Between 25 and 45% of extracranial schwannomas occur in the head and neck region. Within the cranial vault, they are most commonly located at the internal acoustic meatus arising from the vestibular nerves. They are uncommon in the external auditory canal [1]. To the best of our knowledge only 10 cases have been reported in the international literature [1–10]. Chondroid metaplasia, which is seen in our case, has not been reported so far.

We report the first case of schwannoma with chondroid metaplasia in the external auditory canal with the aim of shedding more light on this tumor in this exceptional location, and on the fact that schwannoma should be considered in the differential diagnosis of benign or malignant tissue masses involving the external ear canal, although, in this location, the clinical and radiological findings are somewhat nonspecific.

## Case presentation

**Fig. 1:**
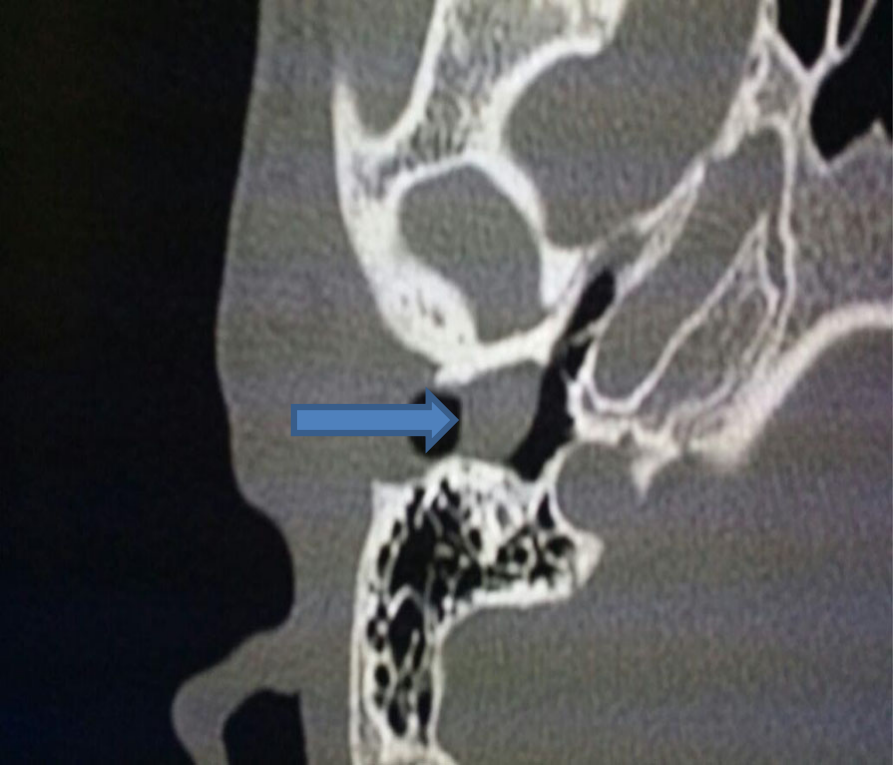
A computed tomography scan of the temporal bone showing a well-circumscribed, soft tissue mass narrowing most of the external auditory canal (arrow) with no bone erosion and no middle ear or mastoid involvement

A 22-year-old white man presented with slowly developing right-side hearing loss over 4 years without external otitis. He has no medical history. His occupation is a student. He did not smoke tobacco or consume alcohol. No familial genetic disorder was found. On admission, his blood pressure was 11/6 mmHg, heart rate was 70 beats/minute, and body temperature was 36.5 °C. A physical examination revealed a pale and firm mass arising from the inferior wall of his right external auditory canal that totally filled the external auditory canal. A neurological examination did not show any lesions in the central or peripheral nervous systems. Laboratory analyses on admission are shown in Table 1. A computed tomography (CT) scan of the temporal bone demonstrated a well-circumscribed, soft tissue mass narrowing most of the external auditory canal (Fig. 1). The mass arose from the inferior canal wall at the cartilaginous portion of his external auditory canal with no bone erosion and no middle ear or mastoid involvement.

**Table 1:** Laboratory data on admission

Hematology	
WBC	7.2 × 10^9^/L
RBC	4.34 × 10^12^/L
Hb	15.2 g/L
Ht	42%
MCV	90.6 fL
PTL	210 × 10^9^/L
Biochemistry	
TP	80.5 g/L
CRP	2 mg/L
Urea	0.3 g/L
Cr	7 mg/L

*Cr* creatinine, *CRP* C-reactive protein, *Hb* hemoglobin, *Ht* hematocrit, *MCV* mean corpuscular volume, *CRP* C-reactive protein++, *PTL* platelets, *RBC* red blood cells, *TP* total protein, *WBC* white blood cells

Our patient underwent, under local anesthesia, an excisional biopsy of the mass via the meatus.

**Fig. 2:**
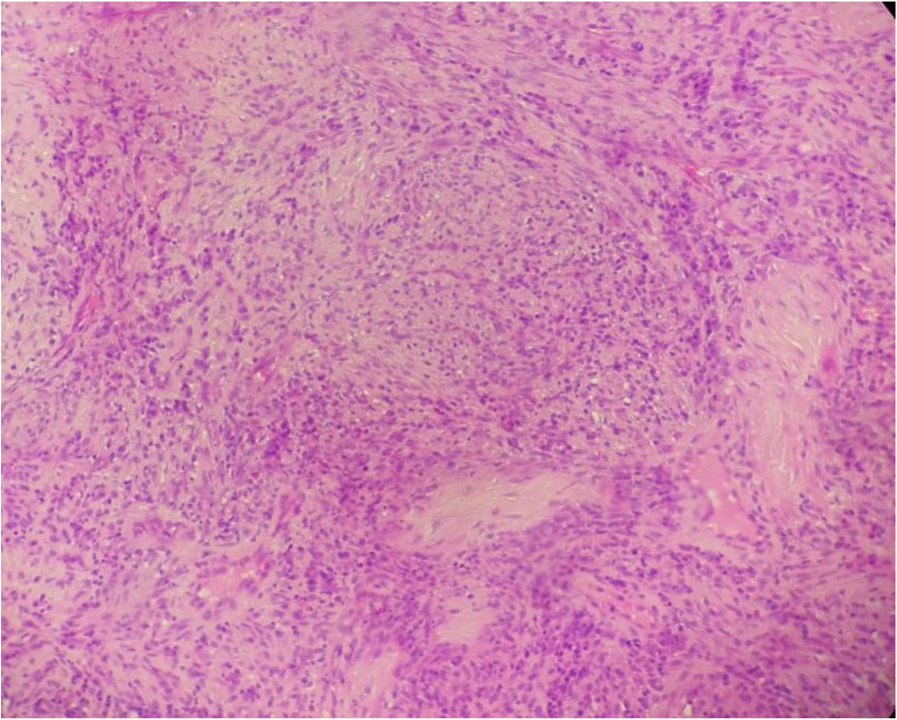
On histology the tumor was composed of spindle cells arranged in interlacing fascicles. Hematoxylin and eosin stain × 100

**Fig. 3:**
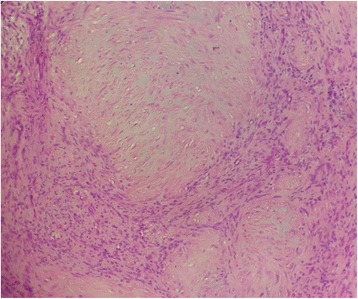
Chondroid metaplasia. Hematoxylin and eosin stain × 400

On histology, the tumor was composed of spindle cells arranged in interlacing fascicles. Focally nuclear palisading was seen. These cells had an abundant eosinophilic cytoplasm and a regular-shaped nucleus without anisokaryosis (Fig. 2). The mitotic ratio was approximately 6 mitoses per 10 high-power field. A chondroid metaplasia (Fig. 3) was seen and thick-walled blood vessels.

**Fig. 4:**
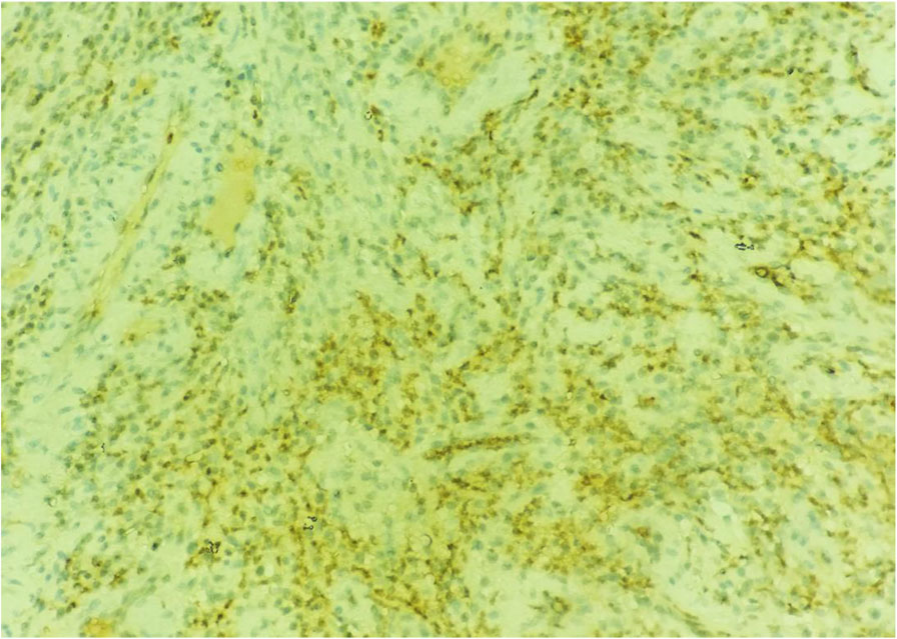
At immunohistochemistry, tumor cells stain strongly for PS100, × 400

At immunochemistry, tumor cells were strongly stained with PS100 (Fig. 4).

The diagnosis of schwannoma with chondroid metaplasia was made.

The mass was completely removed. The tympanic membrane was intact and normal. Our patient’s postoperative period was uneventful. After 8 months there were no signs of local recurrence or narrowing of his external auditory canal.

## Discussion

Schwannomas of the head and neck are common, and are mostly seen arising from the internal acoustic meatus. In the head and neck, they are commonly seen in association with large nerve trunks. Those arising from the external auditory canal are very rare [3]. Most of the extracranial schwannomas in the head and neck originate from cutaneous or muscular branches of the cervical or brachial plexus. Cutaneous sensory nerves that are covered by Schwann cells, from which schwannoma may originate, supply the external auditory meatus and canal. In the present case the tumor was located mainly at the inferior canal wall, which was supplied by the auricular nerve [1].

Only a few cases were reported in the literature. The range of ages in these cases was 18 to 59 years and most patients presented with a mass in their external auditory canal with or without progressive hearing loss. Only one case was discovered by chance during a stapedectomy for otosclerosis [2]. In all the cases a CT scan was performed and it showed a well-circumscribed, soft tissue mass narrowing the external auditory canal. An excisional biopsy was done by transmeatal approach or postauricular approach. The diagnosis was made by histology and no relapses were reported after surgery.

The clinical presentation of schwannoma is usually a slow growing and asymptomatic mass. In the external auditory canal the clinical presentation may appear as recurrent external otitis and a mild conductive hearing loss secondary to obstruction of the canal from the tumor mass [1]. Neurogenic symptoms such as pain or paresthesia are uncommon [5].

Schwannomas are encapsulated and therefore they can be easily dissected from the surrounding tissues. The erosion of the bony canal wall was reported in one case [3].

A differential diagnosis should be made with respect to a number of other soft tissue neoplasms such as fibroma, chondroma, and leiomyoma. A definitive diagnosis should be based on the histological and immunohistochemical findings.

On histologic examination, the tumor is characterized by streams of elongated spindle cells, with the elongated nuclei often arrayed in a palisade pattern. Areas consisting of a thick concentration of cells are called Antoni type A (Verocay body), whereas those in which the cells are loose and irregularly arranged are called Antoni type B. A positive S-100 protein is indicative of Schwann cell origin. Immunohistochemical staining usually shows positive staining for S-100 protein and negative for desmin and smooth muscle actin (SMA).

Schwannomas should be differentiated from other spindle cell tumors such as neurofibroma, leiomyoma, and desmoplastic melanoma. Neurofibromas are not encapsulated and they lack the Antoni-A and Antoni-B pattern. They are usually multicentric, which is an important clinical distinction from schwannomas, and may be accompanied by a special entity called von Recklinghausen’s disease. Leiomyomas are positive for SMA and negative for S-100 while desmoplastic melanomas are positive for S-100 but lack the Antoni-A and Antoni-B pattern.

Radiologic imaging by CT shows schwannomas to be well-circumscribed, homogenous masses that enhance with contrast. CT is also mandatory to rule out the extension of a schwannoma from other temporal bone sites (middle ear, mastoid, or internal auditory canal) to present as an external ear canal mass [4]. A CT scan is very useful in making a decision about the extent of the lesion, integrity of the tympanic membrane, and the type of surgical approach [5].

Treatment is complete excision of the tumor via either transmeatal or post-aural approach. The choice of approach will depend on tumor size, location, and relation to surrounding structures [3].

When complete excision is performed local recurrence is rare [1]. A transmeatal approach was performed in the present case and a good cleavage plane provided an en bloc resection with preservation of surrounding structures [5].

## Conclusion

Schwannoma should be considered in the differential diagnosis of benign or malignant tissue masses involving the external ear canal, although, in this location, the clinical and radiological findings are somewhat nonspecific and rare.
